# Supplementary material for: AlphaFold as a Prior: Experimental Structure Determination Conditioned on a Pretrained Neural Network
Source: bioRxiv. 2025 Mar 11:2025.02.18.638828. Originally published 2025 Feb 21. Preprint. [Version 2] doi: 10.1101/2025.02.18.638828 (PMC11870471; doi:10.1101/2025.02.18.638828)
Supplement: Supplement 1 [file NIHPP2025.02.18.638828v2-supplement-1.pdf]

bioRxiv preprint doi: https://doi.org/10.1101/2025.02.18.638828; this version posted March 11, 2025. The copyright holder for this preprint (which was not certified by peer review) is the author/funder, who has granted bioRxiv a license to display the preprint in perpetuity. It is made available under aCC-BY 4.0 International license.

# Supplementary Table

| Code | n_res | TM_score | Spacegroup | Resolution (Å) | RMSD(AF2, deposited) (Å) |
|---|---|---|---|---|---|
| 7E3Z | 261 | 0.974 | P 1 21 1 | 1.45 | 1.29 |
| 7VNX | 223 | 0.996 | P 61 | 1.80 | 0.75 |
| 7DNT | 250 | 0.990 | P 21 21 2 | 2.50 | 1.74 |
| 7O51 | 207 | 0.994 | P 41 21 2 | 2.20 | 0.90 |
| 7TFQ | 292 | 1.000 | P 21 21 2 | 1.75 | 0.48 |
| 7RAW | 251 | 0.975 | I 21 3 | 2.10 | 0.89 |
| 7FIU | 293 | 0.943 | P 21 21 21 | 1.84 | 1.77 |
| 7SEZ | 221 | 0.990 | C 2 2 21 | 1.70 | 1.75 |
| 7RM7 | 228 | 0.938 | P 21 21 21 | 1.03 | 0.54 |
| 7QDV | 100 | 0.994 | P 43 21 2 | 1.90 | 0.84 |
| 7TRW | 217 | 0.994 | P 62 | 2.28 | 1.96 |
| 7T26 | 144 | 0.999 | P 41 21 2 | 1.14 | 0.67 |
| 7S3L | 269 | 0.997 | P 43 21 2 | 2.60 | 1.56 |
| 7DMS | 94 | 0.424 | P 21 21 21 | 1.96 | 0.56 |
| 7AOJ | 179 | 0.910 | P 61 | 1.63 | 1.60 |
| 7U2R | 245 | 0.999 | P 61 2 2 | 1.85 | 0.54 |
| 7KZH | 202 | 0.979 | P 3 2 1 | 2.49 | 0.62 |
| 7EJG | 237 | 0.998 | P 61 | 1.68 | 1.11 |
| 7T7Y | 154 | 0.990 | P 2 21 21 | 1.81 | 0.68 |
| 7EDC | 249 | 0.989 | C 1 2 1 | 1.95 | 1.46 |
| 7EYJ | 95 | 0.999 | P 62 | 1.38 | 0.64 |
| 7RPY | 256 | 0.957 | P 32 2 1 | 1.67 | 0.84 |
| 7ECD | 272 | 0.997 | I 41 | 2.60 | 1.31 |
| 7UNN | 260 | 0.902 | P 21 21 21 | 1.45 | 0.58 |
| 7TBS | 238 | 0.945 | I 2 2 2 | 1.96 | 0.62 |

**Table 1** Table of high-resolution crystallographic benchmark dataset used for ROCKET. n_res indicates the number of residues.

bioRxiv preprint doi: https://doi.org/10.1101/2025.02.18.638828; this version posted March 11, 2025. The copyright holder for this preprint (which was not certified by peer review) is the author/funder, who has granted bioRxiv a license to display the preprint in perpetuity. It is made available under aCC-BY 4.0 International license.

# Supplementary Figures

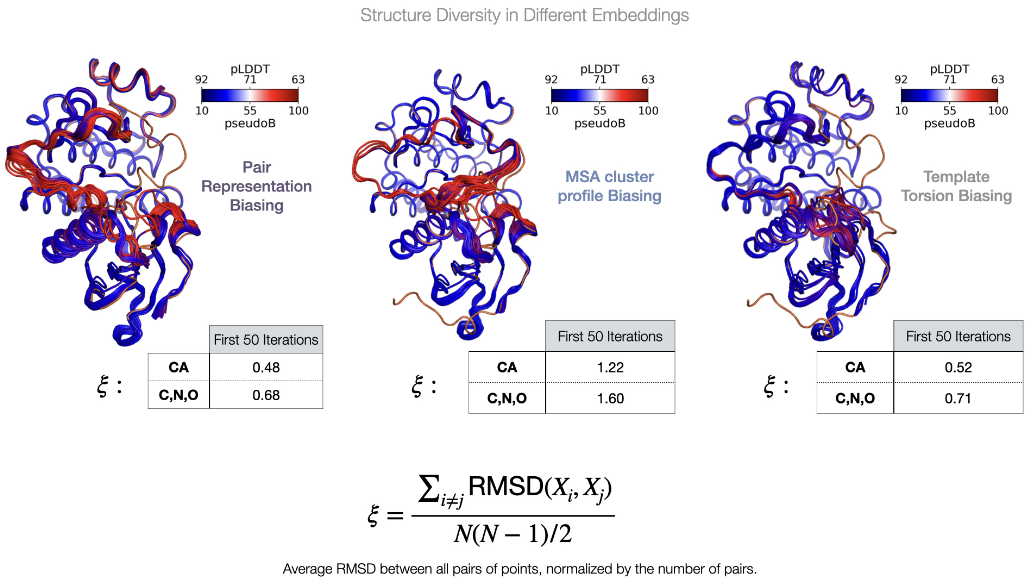


**Fig. S1 ROCKET Optimization in Different AlphaFold2 Embeddings.** We performed data-guided inference-time optimization, as described in the main text, in alternative embeddings. We found that optimizing MSA cluster profiles provides the highest structural diversity along search trajectories. We calculated an average RMSD ($\xi$) between all pairs of structures, normalized by the number of pairs for the first 50 structures in a ROCKET phase 1 refinement run. Diversity is much higher when biasing the MSA cluster profile ($\xi = 1.22$ versus $\xi = 0.48$ and $\xi = 0.52$ when biasing the pair and template torsion representations, respectively).

bioRxiv preprint doi: https://doi.org/10.1101/2025.02.18.638828; this version posted March 11, 2025. The copyright holder for this preprint (which was not certified by peer review) is the author/funder, who has granted bioRxiv a license to display the preprint in perpetuity. It is made available under aCC-BY 4.0 International license.

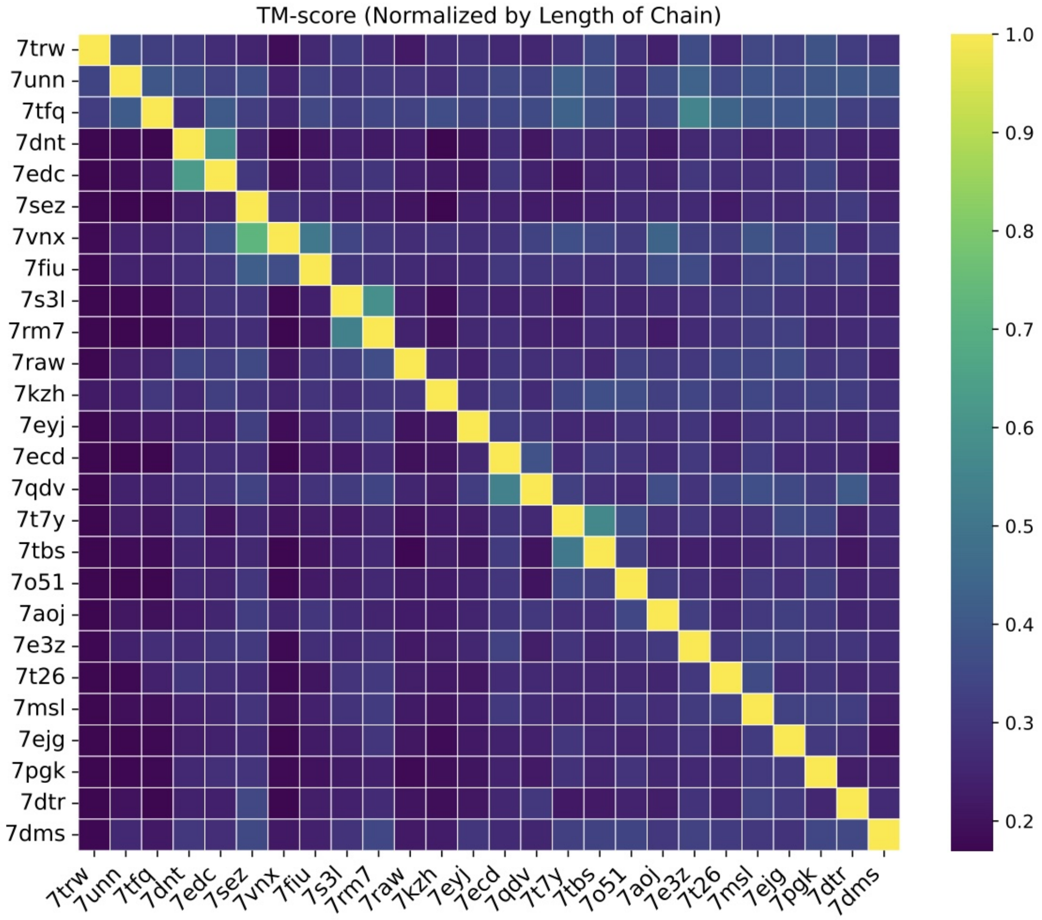


**Fig. S2 Fold Diversity in High-Resolution Benchmark Dataset.** Template Modeling (TM) scores for the 27 structures present in our crystallographic benchmark dataset, identified through their PDB ID. The TM-score evaluates how well two structures align in three-dimensional space, independent of sequence identity, with TM-score closer to 1 indicating very high similarity in the two folds. The low TM-scores between structures reflect their fold diversity. These structures were all released after the training of the AF2 weights used here and were solved by the single-wavelength anomalous diffraction (SAD) method, suggesting that it was challenging to find structural homologs in the PDB for molecular replacement. We therefore expect limited leakage from the original AF2 training set.

bioRxiv preprint doi: https://doi.org/10.1101/2025.02.18.638828; this version posted March 11, 2025. The copyright holder for this preprint (which was not certified by peer review) is the author/funder, who has granted bioRxiv a license to display the preprint in perpetuity. It is made available under aCC-BY 4.0 International license.

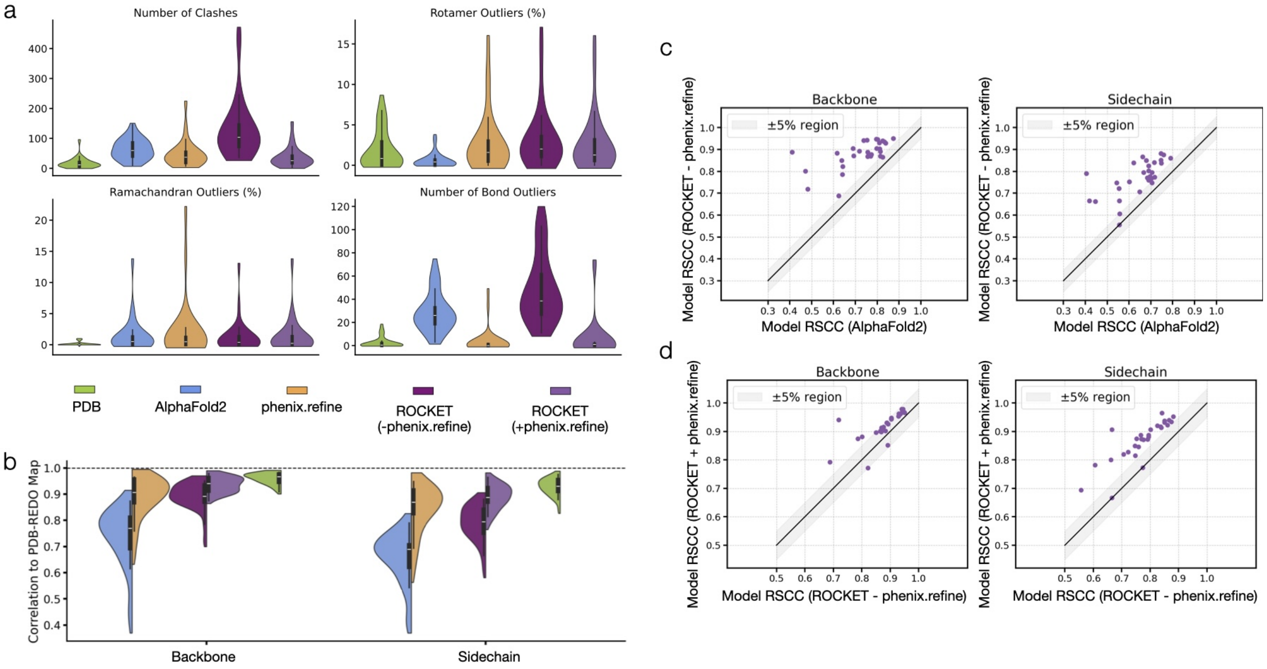


**Fig. S3 Refinement Results for High-Resolution Crystallographic Datasets.** (a) Geometric validation after refinement by different methods, carried out using MolProbity [69] for the 27 high-resolution crystallographic test cases. (b) Real-space Pearson correlation coefficient (RSCC) values for backbone and sidechains across initial AF2 models, phenix.refine, ROCKET refinements without or with the phenix.refine step. All residues were included, including those for which AF2 and ROCKET report low confidence. RSCC values for human-deposited models are also shown. Note that PDB-REDO maps are expected to have favorable phase bias for these models, which will artificially increase reported RSCC. (c-d) Breakdown of the incremental RSCC improvement from the initial AF2 prediction to the ROCKET structures (c), and before and after the phenix.refine step (d).

bioRxiv preprint doi: https://doi.org/10.1101/2025.02.18.638828; this version posted March 11, 2025. The copyright holder for this preprint (which was not certified by peer review) is the author/funder, who has granted bioRxiv a license to display the preprint in perpetuity. It is made available under aCC-BY 4.0 International license.

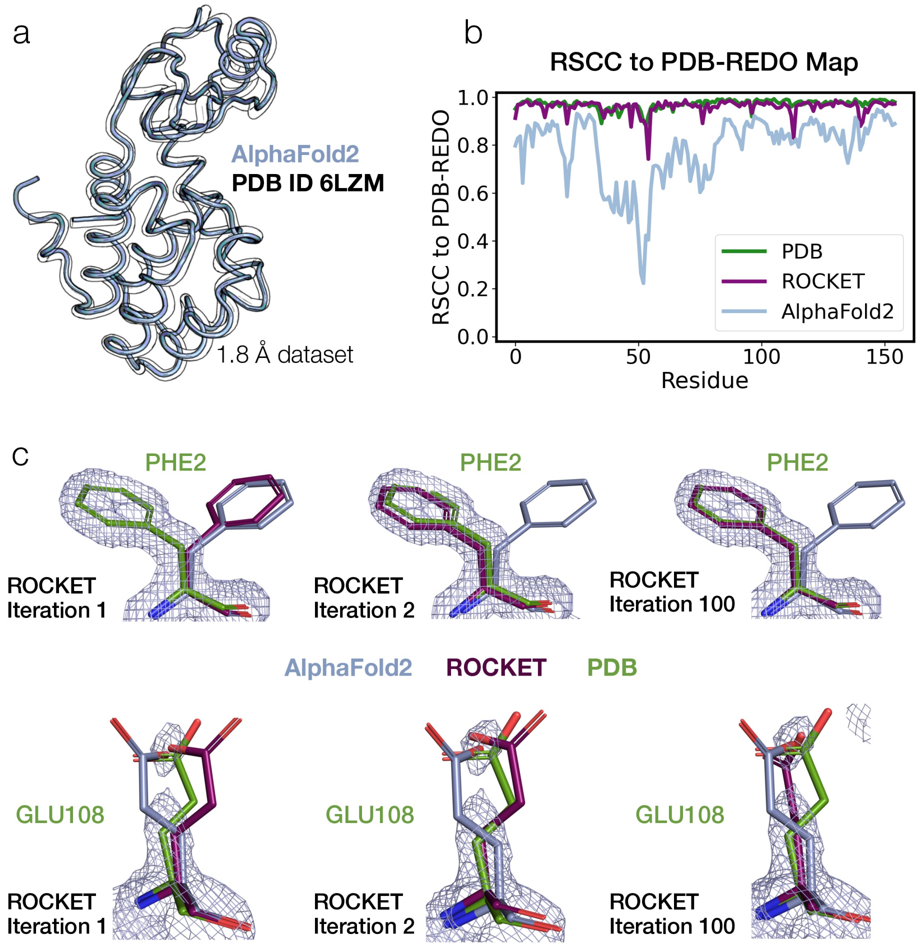


**Fig. S4 MSA Cluster Profile Fine-Tuning Enables Both Backbone and Sidechain Refinement.** Example of ROCKET refinement for a bacteriophage T4 lysozyme dataset (related PDB ID 6LZM), which differs from the AF2 prediction, particularly in the orientation of its upper domain (a). The PDB REDO structure is shown in transparent outlines. (b) ROCKET can refine the prediction to the same quality as the deposited structure. (c) When the data support a clear conformation (*e.g.*, for PHE2), ROCKET is able to find different rotameric configurations and gradually fine-tune the sidechain position. For GLU108, where the data are noisy, ROCKET explores different conformations and, in this case, settles for placing a carboxylate oxygen atom in the available density.

bioRxiv preprint doi: https://doi.org/10.1101/2025.02.18.638828; this version posted March 11, 2025. The copyright holder for this preprint (which was not certified by peer review) is the author/funder, who has granted bioRxiv a license to display the preprint in perpetuity. It is made available under aCC-BY 4.0 International license.

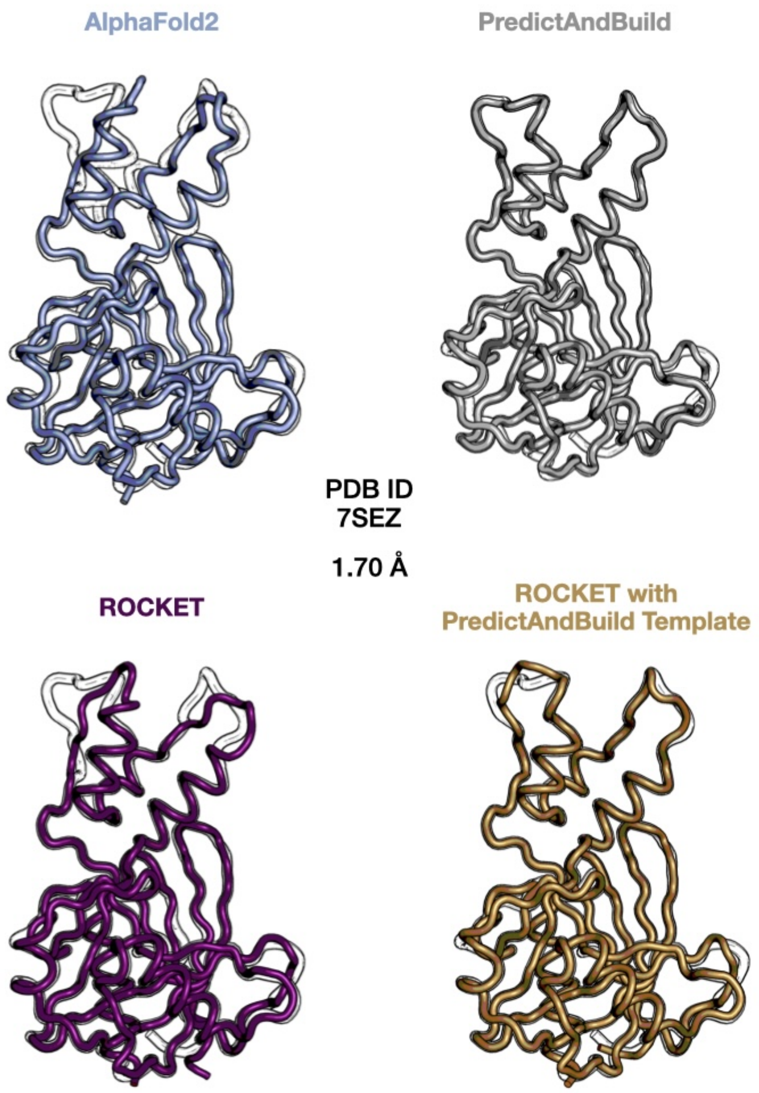


**Fig. S5 Combining ROCKET with PredictAndBuild.** We discuss in the main text and further in Fig. S9 that ROCKET can struggle to flip small loops containing long sidechain residues. The refinement of the Vaccinia Virus decapping enzyme D9 (PDB ID 7SEZ) is an example of this, where ROCKET is unsuccessful in modeling two small loops in the upper domain, while PredictAndBuild converges to the correct backbone. We show the complementarity of the two approaches by running ROCKET with the PredictAndBuild structure provided as a template during OpenFold inference and improving the final output.

bioRxiv preprint doi: https://doi.org/10.1101/2025.02.18.638828; this version posted March 11, 2025. The copyright holder for this preprint (which was not certified by peer review) is the author/funder, who has granted bioRxiv a license to display the preprint in perpetuity. It is made available under aCC-BY 4.0 International license.

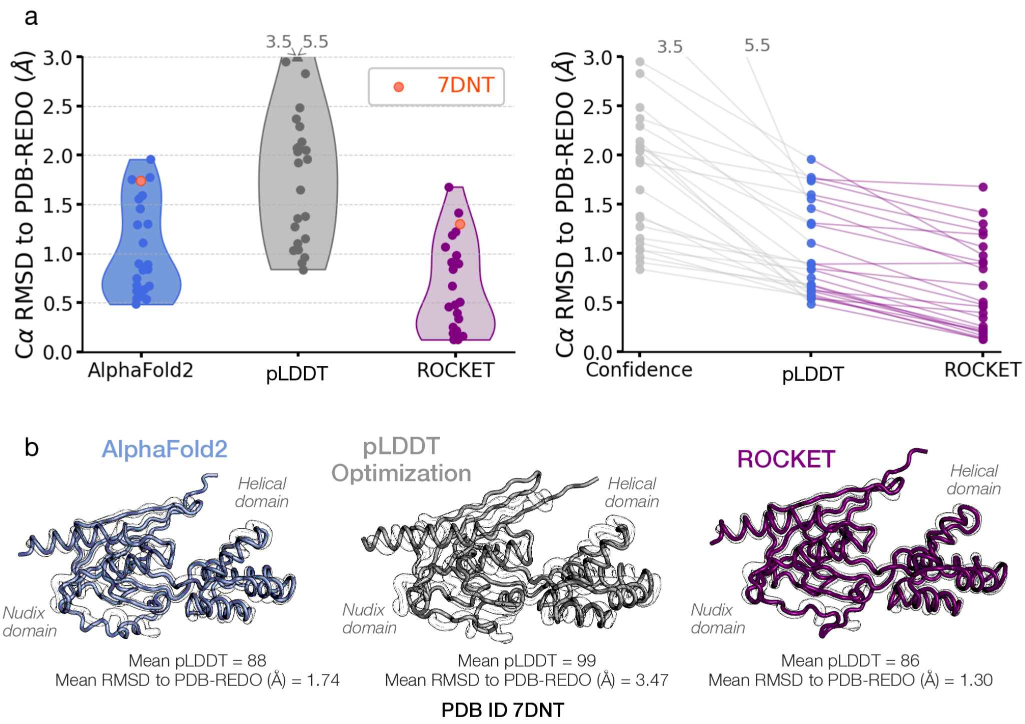


**Fig. S6 Comparison with Confidence Maximization**. To assess the added value of experimental data integration, we compare ROCKET's inference-time optimization to the results that can be obtained by maximizing AF2 model confidence. AF2 model confidence maximization has previously been used to explore alternate conformations for a given sequence, especially in the context of protein design. Depending on the task, different studies have optimized AF2's local confidence metric (predicted local-distance difference test – pLDDT) [56–58], its confidence of relative positioning between residues (predicted aligned error matrix – PAE matrix) [56], or interface confidence for complexes [36, 58]. We conducted a search in the MSA cluster profile space that maximizes pLDDT and found that AF2 does not easily produce experimentally observed conformations without further experimental information. (a) Cα RMSD values between PDB-REDO models and structures from standard AF2 inference, pLDDT maximization, and ROCKET's data-likelihood maximization. ROCKET consistently improves the match between AF2 predictions and experimental structures, while pLDDT maximization alone does not achieve comparable accuracy. (b) Example of the viral mRNA-decapping enzyme g5rp (PDB ID 7DNT) illustrating ROCKET's refinement capability. The initial AF2 prediction shows domain misalignment and secondary structure inaccuracies compared to the experimentally resolved conformation. ROCKET refines these discrepancies, while pLDDT maximization does not converge to the correct structure.

bioRxiv preprint doi: https://doi.org/10.1101/2025.02.18.638828; this version posted March 11, 2025. The copyright holder for this preprint (which was not certified by peer review) is the author/funder, who has granted bioRxiv a license to display the preprint in perpetuity. It is made available under aCC-BY 4.0 International license.

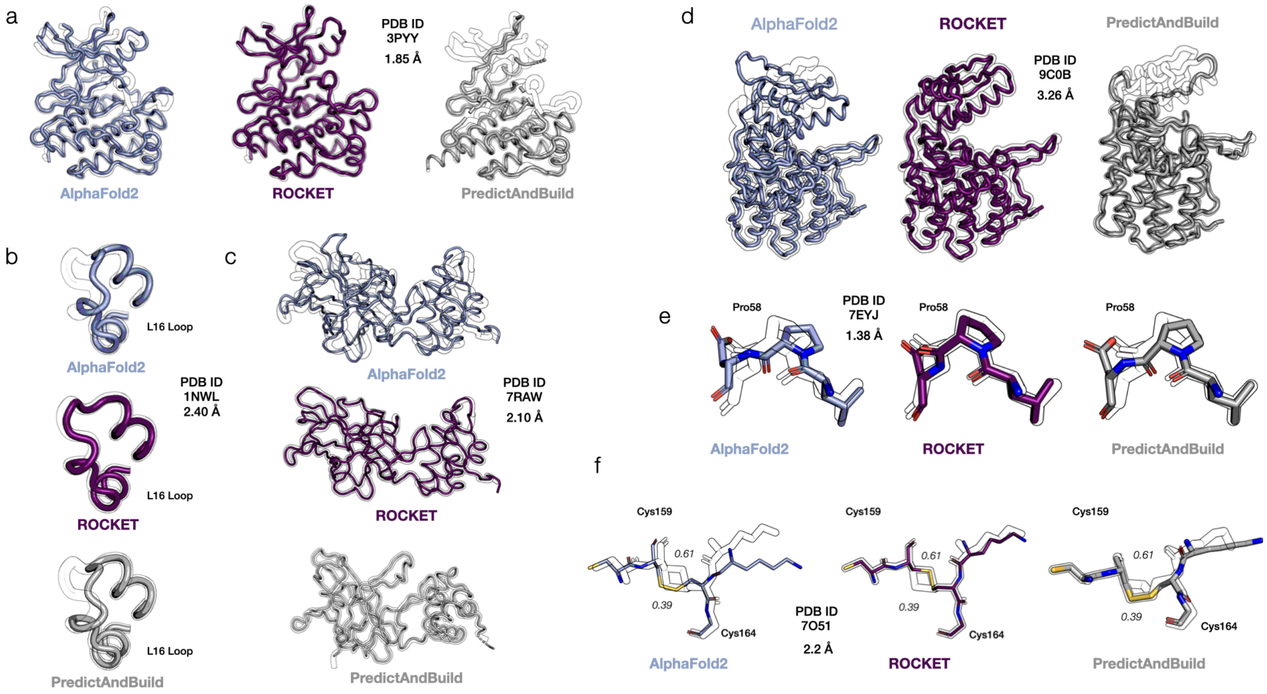


**Fig. S7 ROCKET Overcomes Structural Barriers Inaccessible to PredictAndBuild.** Showing the same examples as in Fig. 3 of the main text, but with the relevant PredictAndBuild refinements. PDB-REDO models are shown in transparent outlines. PredictAndBuild often chops low-confidence regions and flexible loops (see (a) and (d)), leaving them for manual rebuilding. ROCKET can handle these automatically. Two examples of bond rearrangements applied by ROCKET that PredictAndBuild does not access are also shown. The first is a peptide flip in the refinement of *E. coli* nucleoside phoshorylase (PDB ID 7YEJ, (e)). The second is a switch in a disulphide bond in the structure of thaumatin from *T. daniellii* (PDB ID 7AOJ, (f)). Two alternate conformations are present in the deposited structure, with refined occupancies of 0.39 and 0.61. ROCKET builds the conformation with highest occupancy. Both ROCKET and PredictAndBuild can tackle domain rearrangements (c) through separate alignment of the two domains to the data.

bioRxiv preprint doi: https://doi.org/10.1101/2025.02.18.638828; this version posted March 11, 2025. The copyright holder for this preprint (which was not certified by peer review) is the author/funder, who has granted bioRxiv a license to display the preprint in perpetuity. It is made available under aCC-BY 4.0 International license.

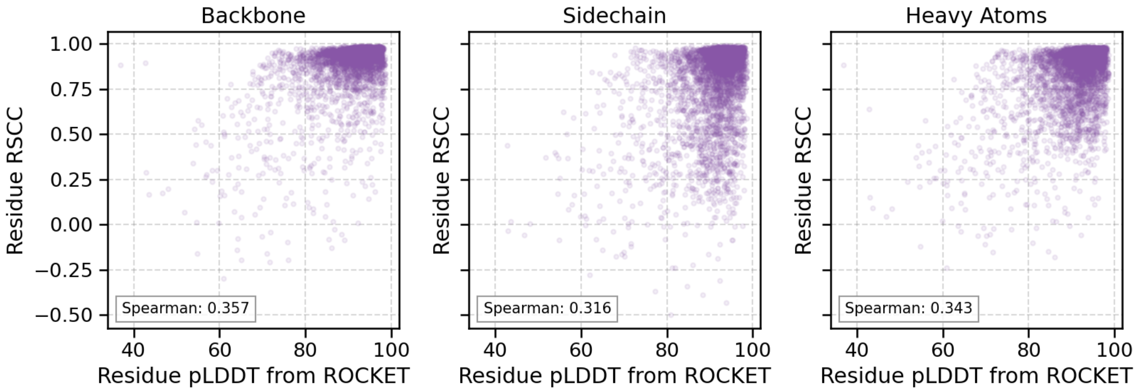


**Fig. S8 Relationship between ROCKET pLDDT and Fit to Experimental Density.** Residue pLDDT at the end of ROCKET refinement for all 27 high resolution test cases are plotted against real-space correlation coefficients (RSCC) between the map calculated from the ROCKET model and the PDB-REDO map. Results are broken down by different atom types.

bioRxiv preprint doi: https://doi.org/10.1101/2025.02.18.638828; this version posted March 11, 2025. The copyright holder for this preprint (which was not certified by peer review) is the author/funder, who has granted bioRxiv a license to display the preprint in perpetuity. It is made available under aCC-BY 4.0 International license.

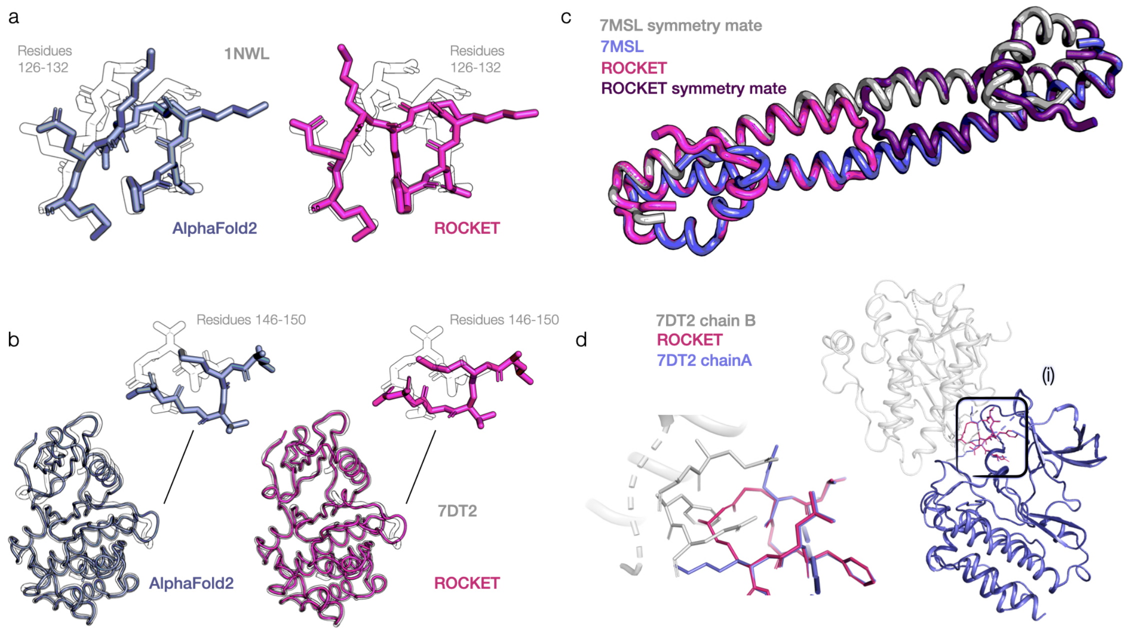


**Fig. S9 Current Shortcomings in ROCKET Model Building.** (a-b) We notice that ROCKET can fail to flip small loops (3-4 residues in length) that contain long sidechain residues. (c-d) Due to OpenFold's lack of awareness of crystal contacts, ROCKET may struggle to converge to certain lattice-dependent conformations (PDB ID 7SML, a dimer with crystallographic symmetry), or to account for the presence of another chain in the asymmetric unit (PDB ID 7DT2).

bioRxiv preprint doi: https://doi.org/10.1101/2025.02.18.638828; this version posted March 11, 2025. The copyright holder for this preprint (which was not certified by peer review) is the author/funder, who has granted bioRxiv a license to display the preprint in perpetuity. It is made available under aCC-BY 4.0 International license.

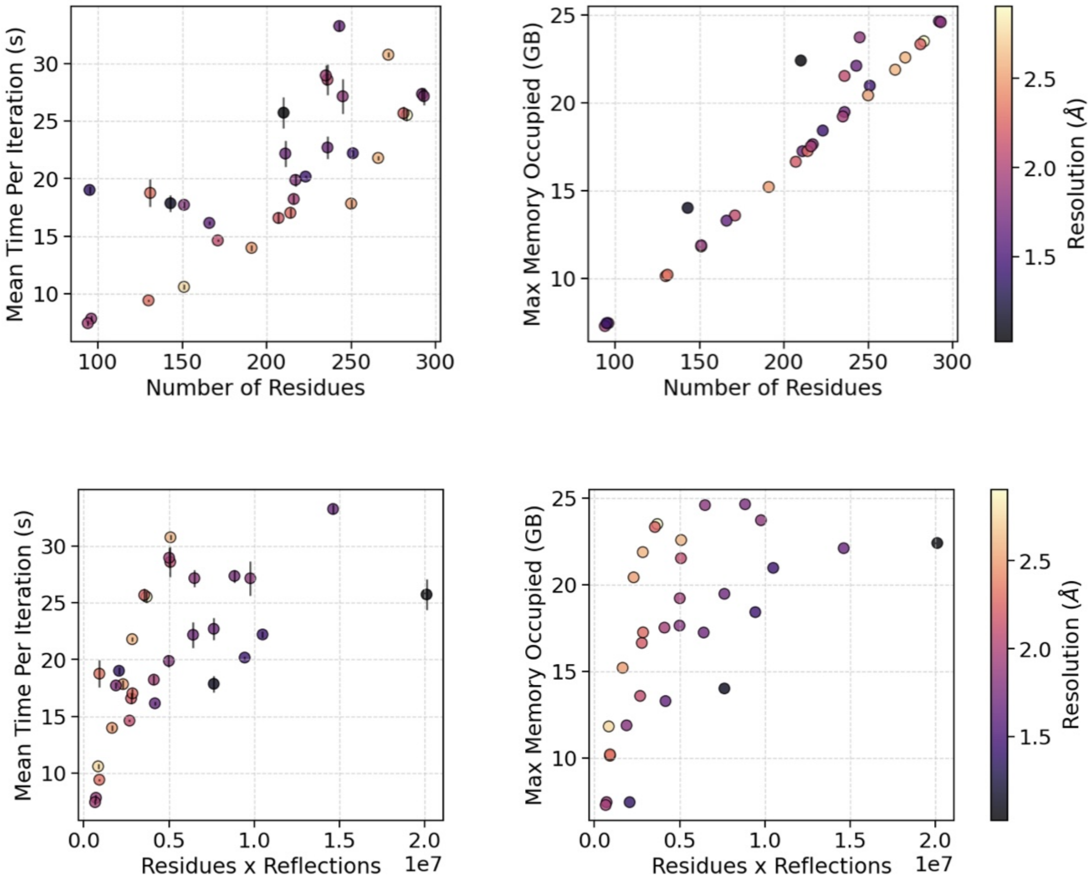


**Fig. S10** **Memory and Computation Time Requirements for ROCKET on a Nvidia 40 GB A100 GPU.**

bioRxiv preprint doi: https://doi.org/10.1101/2025.02.18.638828; this version posted March 11, 2025. The copyright holder for this preprint (which was not certified by peer review) is the author/funder, who has granted bioRxiv a license to display the preprint in perpetuity. It is made available under aCC-BY 4.0 International license.

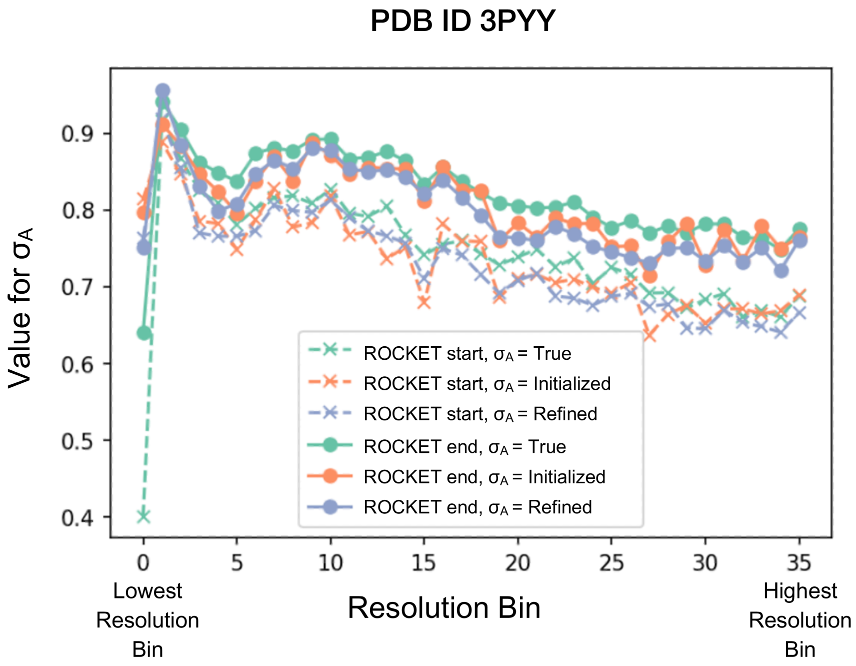


**Fig. S11 Refinement of Crystallographic $\sigma_A$ with the Working Set of Reflections**. We find that refining $\sigma_A$ values with ROCKET using the working set of reflections does not lead to meaningful overfitting. Here we plot $\sigma_A$ values for each resolution bin for the starting (ROCKET start) and final (ROCKET end) iterations of ROCKET refinement for the PDB ID 3PYY dataset of the c-Abl kinase. In orange, we show the initialized values for the iteration and, in blue, the values after the $\sigma_A$ refinement described in Methods. For comparison, we also plot, in green, the $\sigma_A$ values that can be computed using the PDB REDO model phases and that we use as the basis for “true” $\sigma_A$ comparison.

bioRxiv preprint doi: https://doi.org/10.1101/2025.02.18.638828; this version posted March 11, 2025. The copyright holder for this preprint (which was not certified by peer review) is the author/funder, who has granted bioRxiv a license to display the preprint in perpetuity. It is made available under aCC-BY 4.0 International license.

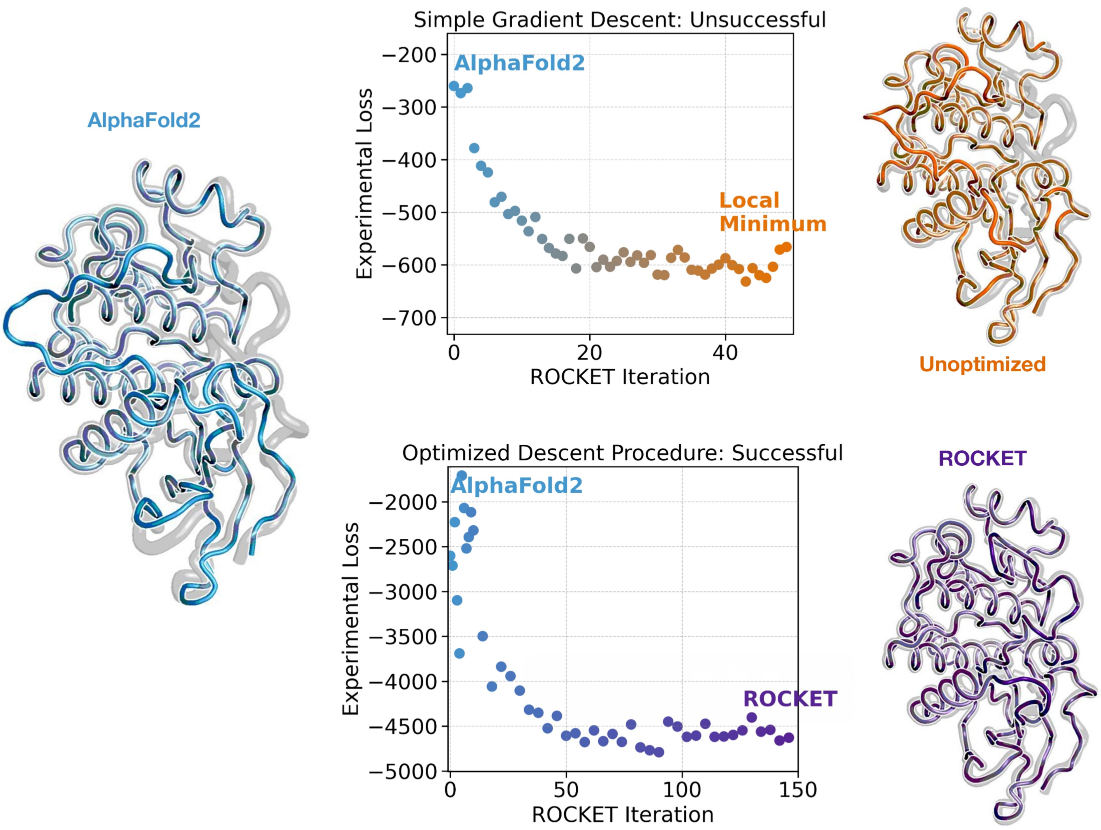


**Fig. S12 Optimization of ROCKET's Gradient Descent Procedure**. Starting from the initial AlphaFold2 prediction for c-Abl kinase, two ROCKET runs are shown for the refinement to a drug-bound crystallographic dataset, where the deposited experimental conformation is shown in gray (PDB ID 3PYY). The top panel displays the refinement results when ROCKET is run with its phase 2 parameters (a low learning rate and an unoptimized gradient descent procedure). The experimental loss (negative LLG score) decreases over the first iterations but plateaus at a local minimum (orange structure), where the main activation loop has not reached the experimental conformation. The bottom panel displays the refinement results when running ROCKET's phase 1, outlined in the Methods. Through this optimized procedure, ROCKET (purple structure) can refine the full backbone so that it matches the experimental conformation.
